# Randomized controlled trials versus rough set analysis: two competing approaches for evaluating clinical data

**DOI:** 10.1007/s11017-014-9283-7

**Published:** 2014-02-20

**Authors:** Tomasz Rzepiński

**Affiliations:** 1 Department of Logic and Methodology of Science, Institute of Philosophy, University of A. Mickiewicz, ul. Szamarzewskiego 89c, 60-569 Poznan, Poland; 2 Department of Biology and Environmental Protection, The Clinical Hospital of Christ Transfiguration, University of Medical Sciences, ul. Długa ½, Poznan, Poland

**Keywords:** Rough set theory, Epistemology, Clinical evidence, Randomized controlled trials, Causality

## Abstract

The present paper deals with the problem of evaluating empirical evidence for therapeutic decisions in medicine. The article discusses the views of Nancy Cartwright and John Worrall on the function that randomization plays in ascertaining causal relations with reference to the therapies applied. The main purpose of the paper is to present a general idea of alternative method of evaluating empirical evidence. The method builds on data analysis that makes use of rough set theory. The first attempts to apply the method show that it is an interesting alternative to randomized controlled trials.

## Introduction

Clearly, no one would deny the fact that clinical decisions need to be empirically justified. The question is, what data can be regarded as a reliable empirical base? With regard to therapeutic decisions, the contemporary approach to this issue has been dominated by evidence-based medicine (EBM), which emerged in the 1990s. This approach postulates a hierarchy of empirical evidence. According to EBM, the most reliable data come from randomized controlled trials (RCTs), i.e., clinical experiments. The data that come from non-experimental studies and from models describing causative mechanisms of therapy in basic sciences are regarded as significantly less reliable. The last two decades have established the conviction that the EBM approach provides the only correct evaluation of the empirical evidence that is required for therapeutic decisions.

The postulate of a hierarchy of empirical evidence plays a crucial part in contemporary medical practice. Although the postulate, being a strong epistemological thesis, requires appropriate justification [1, 2], the supporting argumentation in the literature leaves much to be desired. The main problems concern the privileged epistemological status of the data obtained in RCTs and the relation between the results of these trials and the causal claims that specify clinical events. These objections are my point of departure for developing new methods of obtaining empirical evidence in medicine.

The purpose of this paper is to present a method of obtaining empirical evidence for medical decisions that could be an alternative to RCTs. In this method, an analysis of the clinical data is conducted with the use of rough set theory (RST). The present article will show how applying this method allows one to determine the causal relations of clinical events. The method thus appears to be an interesting alternative to RCT research, in which determining causal relations is frequently a problem.

## Randomized controlled trials and the concept of *probabilistic causality*

In medicine, empirical data can be obtained in various ways. For quite a long time, it was assumed that decisions regarding a therapeutic method ought to be justified by theories and models from the basic sciences. Within this approach, therapeutic decisions were based on knowledge from biochemistry, pharmacokinetics, pathophysiology, and various findings from other basic sciences. They provided only indirect empirical evidence: they were confirmed only insofar as the theories of the basic sciences were also confirmed.

Direct empirical evidence, which concerns the effectiveness of a given therapy, can be obtained via appropriately conducted clinical trials. Three major kinds of trials can be distinguished: case–control studies, cohort studies, and RCTs. While the first two are classified as non-experimental studies, the RCTs are valued highest. In this type of study, a set of patients is randomly divided into an experimental group and a control group. The appraisal of effectiveness consists in comparing the effects of the therapy in the treatment group and the control group. An RCT can be a double-blind study (neither the subject nor the doctor knows whether a real treatment or a placebo is applied) or a single-blind study (either the subject or the doctor does not know whether a real treatment or a placebo is applied).

The development of the EBM approach strengthened the conviction that RCTs provide the most reliable empirical evidence for a therapy’s effectiveness. This assumption is, nevertheless, a strong epistemological thesis. Consequently, it seems advisable to investigate how the assumption is justified. John Worrall identifies four major arguments put forward by the proponents of randomization [1]. Two of these are particularly relevant to our analysis.

The first one concerns the distribution of the confounding factors in the control and experimental group. The uneven distribution of such factors can result in a selection bias. The proponents of RCTs maintain that randomization guarantees an equal distribution of both known and unknown confounding factors in the groups. However, the opponents point out that an equal distribution of the known confounders does not guarantee that randomization will provide an equal distribution of the unknown confounders [1–4].

Another important argument in favor of randomization is formulated by the proponents of probabilistic theories of causality. Here, the basic idea is that probabilistic dependencies must have causal explanations [5]. Such a position is embraced by David Papineau [6]. In his opinion, randomization guarantees that the applied therapy is not itself objectively correlated with any other characteristics that influence the recovery. Consequently, if it is ascertained that in randomization, the probability of recovery (*R*) given the therapy (*T*) is greater than the probability of recovery given the absence of the therapy (*¬T*), i.e.,

$$ P(R/T) > P(R/{\neg }T), $$

then one may also assume that *T* causes *R* [6].

Worrall opposes this argument by considering an example that is based on the following assumptions:

(i) in the case of disease *D*, therapy *T* causes recovery *R*, iff factors *f*_1_, …, *f*_*i*_ appear in patient;
(ii) factors *f*_1_, …, *f*_*i*_ occur very rarely in the population, and the doctors are not familiar with them;
(iii) in the case of the absence of these factors, therapy *T* causes severe side effect *¬R* in a given patient.
(iv) an RCT has been conducted and randomization has led to an equal distribution of the known and the unknown confounders in the experimental and the treatment groups [7].

Nevertheless, due to the fact that factors *f*_1_, …, *f*_*i*_ occur very rarely in the population, it has been ascertained that:

$$ g(R/T) < g(R/{\neg }T),{\kern 1pt} \quad {\text{where}}\;g\;{\text{is}}\;{\text{frequency}}. $$

In this example, Papineau would suggest that the researchers should draw the conclusion that therapy *T* is the cause of *¬R* [6]. However, such a conclusion would be wrong, according to Worrall, because of assumption (i). Worrall admits that the example is unrealistic, but it nonetheless allows one to show that randomization cannot provide a basis for causal claims.

In what follows, it will be argued that Worrall’s example is not different from various cases that occur in actual medical practice. First, however, the example supports an observation that will prove crucial for my further consideration:

### *S*_1_:

Establishing the causal relations between events *T* and *R* requires identifying factors *f*_1_, …, *f*_*i*_, whose occurrence together with *T* will determine the occurrence of *R*.

Such an account of the causal relation has been endorsed by Nancy Cartwright [5]. In her probabilistic theory of causality, Cartwright assumes the following principle for determining causal relations.

$$ CC:\;T\;{\text{causes}}\;R\quad {\text{iff}}\;P(R/T{ \wedge }K_{i} ) > P(R/{\neg }T{ \wedge }K_{i} ), $$

where *K*_*i*_ is a complete causal set for *R* (obviously apart from *T*) and *K*_*i*_ = {*f*_1_, …, *f*_*i*_} [7–9].

When analyzing the issue, Cartwright introduces the concept of the *ideal RCT* [5, 8, 9]. An ideal RCT is characterized as research in which applying the procedure of randomization causes an equal distribution of factors from set *K*_*i*_ in the treatment and the control group. However, the problem is—as Cartwright notices herself—that it is not clear how close the real RCT is to the ideal one [5, 10].

One more issue regarding the CC principle needs to be pointed out. The confounding factors that appear in set *K*_*i*_ can have different values. Consequently, certain values of the factors confounding therapy *T* may cause *R*, whereas other values of the same factors may cause *¬R*. Randomization ought to secure not only an equal distribution of the confounders in both groups, but also an equal distribution of the values of these confounders. Conducting ideal RCT would show the essential advantage of randomization: it allows one to learn causal relations without having to know what the possible confounding factors actually are, and how they ought to be characterized [5].

The advantage that Cartwright points to is, nevertheless, rather illusive. The lack of knowledge about the confounding factors is the major obstacle in preparing a deterministic decision algorithm (DDA) for the particular patients. At most, one can hope that the RCT’s experimental population was a representative sample of the target population, and that the findings of the RCT will also be valid in the target population.^1^ Yet, in this case, the findings concerning the target population are merely statistical. One can, for example, ascertain the absolute risk reduction (ARR) for an unfavorable clinical event. Once the value is determined, the number needed to treat (NNT), i.e., the number of patients that have to be treated if *one* of them is to be treated successfully, may be specified. Still, what is the value of such information? Consider the following example. If on the basis of an RCT it is established that NNT = 100, then this means that only 1 patient out of 100 will benefit from the therapy. Without knowing the effect that the confounding factors have on the therapy, one cannot ascertain what the benefits will be for any particular patient.

The fact that we have a probabilistic theory of causality at our disposal is not of much help if we do not know which patients will benefit from the therapy and which will not. In order to establish that, we need to specify the set of confounding factors and the range of values in which *T* causes *R*. Lack of knowledge about the confounding factors can hardly be treated as an advantage of randomization. Neither does randomization seem to be perceived in this way by clinicians. In clinical trials, there is a clear tendency to narrow down the range of the population qualified for trials due to the occurrence of certain known confounders. Moreover, one should also bear in mind that clinical trials are frequently subject to further subgroup analysis, in which the population under investigation is divided with regard to certain special factors. Such analyses point to a tendency that is crucial in clinical practice: from a clinical perspective it is important to specify what factors (and their values) qualify the patient for a given therapy.

Summing up, we may say that randomization is conducted not because we are not interested in the specification of the confounding factors, but rather because we have no knowledge of these factors. By applying it, we hope that randomization will enable us to make certain approximations about the effectiveness of the therapy, precisely when no knowledge of these factors is available. These approximations are, however, purely statistical. In medical practice, deterministic knowledge, i.e., knowledge that specifies for which particular patients applying a given treatment will bring about the expected benefits, is highly preferred. This kind of knowledge might be the basis for creating deterministic decision algorithms. I will now take a closer look at this issue, using the basic rules regarding the classification procedure.

## Classification and algorithms of therapeutic decision making

Assume there is a set *U* of certain objects. Making the classification of the elements that belong to set *U*, one can distinguish its subsets in such a way that the following conditions are met:

(i) the intersection of any subsets is an empty set, i.e., none of the objects can be an element of more than one subset;
(ii) every object from set *U* must be an element of one of the subsets, i.e., the union of the subsets equals *U*;
(iii) none of the subsets can be empty.

A simple example can be considered in which *U* = {*a*_1_, *a*_2_, *a*_3_, *a*_4_}. By distinguishing subsets *A*_1_ = {*a*_1_, *a*_2_}, *A*_2_ = {*a*_3_}, and *A*_3_ = {*a*_4_}, the criteria for classifying the elements in set *U* are met because (i) the subsets share no elements, (ii) all the elements in set *U* are elements of one of the subsets, and (iii) none of the subsets is empty.

One can now observe that the notion of classification can be easily applied to situations regarding therapeutic practice, forming the basis for creating decision algorithms. Suppose that there is a set of patients *U* who have been diagnosed with the same disease *D*. These patients, suffering from the same condition, differ in the values of additional attributes *f*_1_, …, *f*_i_. The values of some of these attributes depend on the patients’ individual characteristics (age, sex, race, etc.), while others can be the result of disease development. One can thus state that the values of attributes *f*_1_, …, *f*_i_ form a patient’s pre-therapy characteristics. Knowing these values, one can classify patients from set *U* according to their pre-therapy characteristics.

Assume, then, that different values of pre-therapy attributes influence the effects of the therapy in particular patients (principle *S*_1_). Some experience a spectacular improvement while others enjoy relatively small therapeutic benefits. Thus, taking into account the effects of the applied therapy, one can make a classification of patients, mapping them to different recovery classes *R*_1_, …, *R*_i_.

In an ideal situation, for each set of patients defined by the values of pre-therapy attributes, one could map exactly one recovery class. In such a situation, *S*_1_ can be expanded as follows:

### *S*_2_:

Establishing a deterministic decision algorithm (DDA) requires specifying the values of the factors *f*_1_, …, *f*_*i*_, whose occurrence together with *T* will determine *R*.

### *DDA:*

For any population A, which is characterized by set of factors *f*_1_, …, *f*_*i*_, the relation *T(A)* → *R(A)* holds true [5, 8].

If such an algorithm could be formed for specific clinical cases, it would enable one to unequivocally determine the rational procedure for making therapeutic decisions.

In RCT research, however, classification of patients is conducted in a different manner from the one described above. It is true that there are two subgroups (the treatment group and the control group),^2^ and that the three conditions of classification (i–iii) are fulfilled. The randomization procedure is supposed to ensure equal distribution of known and unknown confounding factors in the treatment group and the control group. What guarantee is there, however, that such equal distribution will occur? Proponents of randomization argue that the knowledge of equal distribution of known confounding factors increases the probability of equal distribution of unknown factors.

But what notion of probability do they have in mind in this situation? They would surely want the odds of having an equal distribution of unknown factors to have an objective character. Therefore, they would have to accept the frequency concept of probability. However, the knowledge of the known does not increase frequency probability of the occurrence of the unknown. The knowledge of the equal distribution of known confounders does not increase the objective probability of the occurrence of unknown factors, but at most, adds to belief-type probability, understood as the strength of a person’s belief regarding the occurrence of these factors [11, p. 9].

A person’s subjective conviction is not enough, however, to claim that correctly performed randomization guarantees an equal distribution of unknown factors. The problem is that by classifying patients using the procedure of randomization, we give up on attaining a much more valuable cognitive objective, which is determining the effects of confounders on the course of therapy. This objective may be achieved, however, using RST data analysis, which I will now consider.

## Classification and confounding factors in therapy

I now consider how the classification procedure can be used for assessing the influence of confounders. I assume the following:

1. we consider a set of patients U = {*a*_1_,…*a*_8_} diagnosed with disease *D*,
2. *f*_1_, *f*_2_, *f*_3_ are the attributes of the patient before therapy *T* (i.e., condition attributes),
3. attributes *f*_1_ and *f*_2_ take values 0 or 1,
4. attribute *f*_3_ takes values 0, 1, or 2,
5. applying therapy *T* leads to two recovery classes, i.e., *Ω* = {*Y*_1_ *, Y*_2_}.

This means that every patient under the therapy can be characterized by additional attribute *r*, taking values 0 or 1. These can be interpreted in the following way: 0 = patient deterioration, 1 = recovery (*r* can be termed a “decision attribute”).^3^ The values of the attributes for a particular set of hypothetical patients and their outcomes are illustrated in Table 1.

**Table 1:** Values of pre-therapy attributes and their influence on the therapy results

	*f*_1_ *(a*_*i*_ *)*	*f*_2_ *(a*_*i*_ *)*	*f*_3_ *(a*_*i*_ *)*	*r(a*_*i*_ *)*
a_1_	0	1	0	**0**
a_2_	1	0	2	**1**
a_3_	0	0	0	**0**
a_4_	1	0	2	**0**
a_5_	0	1	0	**0**
a_6_	1	1	1	**1**
a_7_	0	1	1	**1**
a_8_	0	0	1	**0**

Each line in Table 1 provides certain information about a given patient. On the basis of this information, one can make two classifications of patients from set *U*. The first classification would be made according to the values of pre-therapy attributes. The second would be made according to the value of attribute *r*, which determines the therapy effects.

In the case of the first classification, the following subsets of patients can be differentiated:

1 $$ X_{1} = \left\{ {a_{1} ,a_{5} } \right\},\;X_{2} = \left\{ {a_{2} ,a_{4} } \right\},\;X_{3} = \left\{ {a_{3} } \right\},\;X_{4} = \left\{ {a_{6} } \right\},\;X_{5} = \left\{ {a_{7} } \right\},\;X_{6} = \left\{ {a_{8} } \right\} $$

Patients in each of these subsets have the same characteristics of pre-therapy attributes: {*f*_1_, *f*_2_, *f*_3_}. In the case of the second classification, the recovery classes are subsets:

2 $$ Y_{1} = \left\{ {a_{1} ,a_{3} ,a_{4} ,a_{5} ,a_{8} } \right\},Y_{2} = \left\{ {a_{2} ,a_{6} ,a_{7} } \right\} $$

Observe that the example I am considering represents Worrall’s “unrealistic” example, as most of the patients do not benefit from the therapy. The therapy is beneficial to only three patients (*a*_2_ *, a*_6_ *, a*_7_). Conducting an RCT would thus only average the results, showing the therapy to be harmful.

There is one problem, however. It should be noted that patients *a*_2_ and *a*_4_ from Table 1 cannot be unequivocally assigned to any recovery class on the basis of the values of the pre-therapy attributes. That is, even among patients who have the same relevant attributes, only some of the patients given the therapy recover. Consequently, one cannot establish a DDA for those future patients *x*_*i*_, whose attribute values will be the same as the attribute values of patients *a*_2_ and *a*_4_. The set {*a*_2_, *a*_4_} must be characterized as a *doubtful region of classification* [12]. It turns out, then, that one does not have complete knowledge of the indications and contra-indications for conducting the therapy. Unfortunately, this is a situation that most doctors are faced with in their practice. How, then, should one make clinical decisions? An attempt to solve this problem can be made on the basis of RST [12–14].

## Recovery classes in the RST data analysis

Therapeutic decisions are based on the knowledge about circumstances in which a given therapy will be beneficial for a patient and circumstances in which it can cause adverse effects. The knowledge of the indications for conducting the therapy is not obviously tantamount to the knowledge of contra-indications for this therapy. Knowing that certain values of pre-therapy attributes are causally relevant to producing positive therapy effects, we cannot infer that their other values constitute contra-indications to conducting the therapy. An additional hindrance to establishing indications and contra-indications is the fact that we do not possess complete knowledge about the effects caused by particular confounders due to the doubtful region of classification—in the discussed example, {*a*_2_, *a*_4_}. Using the empirical information about pre-therapy attributes and the results of treatment from a hypothetical example depicted in Table 1, one can characterize recovery classes with better accuracy. This accuracy can be defined in RST as the accuracy of approximation of a given recovery class by the set of pre-therapy attributes [15].

Characterizing the term *approximation of a given recovery class by the set of pre*-*therapy attributes* one should take into account the following:

- the fact that there are patients who (based on information from Table 1) definitely belong to particular recovery classes,
- the fact that there are patients about whom it is unclear which recovery class they belong.

These latter patients (described in point b, above) are the patients from the doubtful region of classification, i.e., *a*_2_ and *a*_4_ in the hypothetical example.

Based on the findings, one can thus ascertain that the accuracy of approximation for a given recovery class should be inversely proportional to the breadth of the doubtful region of classification, i.e., the larger the set of the patients who cannot be unequivocally assigned to one recovery class based on the values of the pre-therapy attributes, the lower the accuracy of the approximation will be. The accuracy of approximation should, then, increase if fewer patients fall into the doubtful region of classification. This intuition within RST is understood in such a way that the accuracy of approximation is characterized as the ratio of the number of patients who have been definitely assigned to a given recovery class divided by the total number of patients in that recovery class (i.e., the sum of the definitively and the doubtfully classified as either recovering or not recovering) [15]. This description can be illustrated with an example showing the accuracy of approximation for recovery class *Y*_1_.

Based on classifications (1) and (2) it can be said that patients *a*_1_, *a*_3_, *a*_5_, *a*_8_ are definitely correctly assigned to recovery class *Y*_1_ (non-recovery), since Table 1 contains no other patients who would display the same characteristics of pre-therapy attributes and who would belong to a recovery class other than *Y*_1_. The set of these patients can be marked as *P*. It is a four-element set. To this the set of patients from the doubtful region of classification, i.e., {*a*_2_, *a*_4_}, can be added. The resulting set has six elements (it is marked *L*). Thus, the accuracy of approximation of recovery class *Y*_1_ based on the set of attributes *f*_1_, *f*_2_, *f*_3_ is:

3 $$ \alpha (Y1){ = }\frac{card(P)}{card(L)} = 4/ 6= 0. 6 6 $$

Where card(P) and card(L) are the cardinality of set *P* and *L* respectively.

In a similar way, one can calculate the accuracy of approximation for recovery class *Y*_2_. Based on (1) and (2) we can ascertain that patients *a*_6_, *a*_7_ certainly belong to this recovery class. The set of these patients can be marked as *P′*, and the set of patients from the doubtful region of classification, i.e., {*a*_2_, *a*_4_}, can be added to it. The resulting set has four elements (it is marked *L′*). So, the accuracy of approximation of recovery class *Y*_2_ is:

4 $$ \alpha (Y2){ = }\frac{card(P^{\prime})}{card(L^{\prime})} = 2/ 4= 0. 5 $$

It is thus evident that the doubtful region of classification can affect to varying degrees the accuracy of approximation of a given recovery class. One recovery class may be characterized with very high accuracy, while others may be characterized with much lower accuracy. In such a situation, there would be a well determined decision algorithm for a certain group of patients, whereas the prognosis for the remaining patients with different characteristics of pre-therapy attributes could be very inaccurate.

It should be noted that in RCTs, one does not know the precise characteristics of the recovery classes. Acquainting oneself with the results of an RCT, one only learns what the inclusion criteria were for patients in the trial but not what additional factors characterized the patients who recovered or did not benefit from the therapy.

## The causal relevancy of attributes in RST approach

The possibility of specifying the accuracy with which patients are assigned to particular recovery classes also allows one to determine the causal relevancy of particular pre-therapy attributes [16]. The idea is as follows.

If a given pre-therapy attribute is causally significant for the effects of the therapy, then removing it from the Table should reduce the accuracy of approximation for at least one recovery class. This intuition can be illustrated using the example analysed earlier, by removing attribute *f*_2_ from Table 1, and seeing whether the accuracies of approximation in recovery classes *Y*_1_ and *Y*_2_ will change.

After removing attribute *f*_2_, the following classification of patients results on the basis of the values of attributes *f*_1_, *f*_3_.

5 $$ \begin{gathered} {\text{Definitive non - recovery}}:X_{1}^{{\prime }} = \left\{ {a_{1} ,a_{3,} a_{5} } \right\} \hfill \\ {\text{Doubtful non - recovery}}:X_{2}^{{\prime }} = \left\{ {a_{2} ,a_{4} } \right\} \hfill \\ {\text{Definitive recovery}}:X_{3}^{{\prime }} = \left\{ {a_{6} } \right\} \hfill \\ {\text{Doubtful recovery}}:X_{4}^{{\prime }} = \left\{ {a_{7} ,a_{8} } \right\} \hfill \\ \end{gathered} $$

The overall classification of patients as given in (2), above, will not be altered since they belong to recovery classes *Y*_1_ and *Y*_2_.

Based on (5) and (2) one can state that patients *a*_1_, *a*_3_, *a*_5_ are definitely correctly assigned to recovery class *Y*_1_ (non-recovery), because there are no other patients from $X_{1}^{{\prime }}$ who would display the same characteristics of pre-therapy attributes, and who would belong to a recovery class other than *Y*_1_. Patient *a*_8_, however, cannot be correctly assigned to this recovery class. Removing attribute *f*_2_ caused patient *a*_8_ to display exactly the same specifications of pre-therapy attributes as patient *a*_7_ (both now belong to set $X_{4}^{{\prime }}$). The problem is, however, that critical information has been lost. Patient *a*_8_ is assigned to two different classes of recovery. Removing attribute *f*_2_ thus caused an increase in the doubtful region of classification, which must now be specified as {*a*_2_, *a*_4_, *a*_7_, *a*_8_}. This loss of information resulted in lowering the accuracy of the approximation for both recovery classes.^4^ It can, therefore, be inferred that attribute *f*_2_, differentiating pre-therapy specifications of patients *a*_7_ and *a*_8_, is causally relevant to the course of therapy.

As described, RST also allows one to determine relatively easily which pre-therapy attributes are most causally relevant for the therapeutic effects. One can remove successive attributes from the Table, and check the extent to which the accuracy of approximation for particular recovery classes will decrease. The larger the decrease in the accuracy of approximation for a given class, the greater the causal relevancy of the removed attribute. For instance, one should note that in the hypothetical example given in Table 1, the doubtful region of classification would be much greater if one removed attribute *f*_3_ instead of attribute *f*_2_. Consequently, the accuracy of approximation for both recovery classes would also decrease significantly.^5^ Based on this, one can conclude that attribute *f*_3_ is causally more significant for the therapeutic effect than attribute *f*_2_.

## Rough set theory versus subgroup analysis

Does RST offer any advantages over subgroup analysis? A skeptic might argue that the procedure of removing successive attributes is in fact similar to subgroup analysis, which can be conducted after an RCT. In this type of analysis, similarly to RST analysis, one is also looking for factors differentiating the effects of the therapy. Nonetheless, methodologists have raised serious doubts about the validity of conducting subgroup analysis after an RCT [17–19]. Does RST analysis not raise similar doubts?

The difference between the two procedures lies in the assumptions made for the purpose of realizing one’s cognitive objectives. RST data analysis is based on the assumption that it is possibile to evaluate the causal relevancy of particular pre-therapy attributes. Under the working assumptions of an RCT, such evaluation would be superfluous since it is assumed that the treatment group and the control group can be standardized with respect to these attributes.

The differences in the assumptions underlying both procedures affect the way the research goals within them are pursued. RST data analysis allows one to determine the initial value of the accuracy of approximation for particular recovery classes. That is why the subsequent procedure of removing successive attributes allows one to determine their causal relevancy. There is a point of reference, which is the initial value of the accuracy of the approximation. However, in an RCT, one does not have the initial value of the accuracy of approximation. It is assumed, after all, that randomization brought about an equal distribution of the confounding factors. Therefore, subgroup analysis cannot determine the effects of a change in the initial value of pre-therapy attributes. All that subgroup analysis after an RCT can do is to point out statistical correlations. Inference in subgroup analysis about causative factors based on accidental correlations may lead to erroneous or even absurd conclusions, for example, that a given treatment is harmful for people born under the signs of Gemini and Libra [20].

## Identyfing redundant information in an RST approach

An additional advantage of the RST method is the fact that it allows one to determine easily which pre-therapy attributes are not causally relevant to the effects of the therapy. The intuition is simple. The information about the values of pre-therapy attributes allows us to classify patients into outcome classes. Therefore, if removing a given attribute will not change this classification, one can claim that it is not causally relevant to the effects of the therapy. Such an attribute can be termed as a *superfluous attribute* [15].

The possibility of identifying superfluous pre-operational attributes is an important merit of the RST method. The development of new diagnostic techniques (including genetic, biochemical, and imaging tests) makes it possible to obtain more and more information about the condition of patients. These techniques make it possible to diagnose disease in its early stage and to monitor its development. It is common knowledge that patients who have been diagnosed with the same disease can differ with regard to the values of many diagnostic and prognostic factors. Frequently, different values of these factors can be of great importance to the results of therapy. It can also happen, however, that different values of a given attribute exert no impact on the therapy whatsoever. In these cases, the usefulness of a particular factor may be limited solely to the diagnostic process, whereas in the process of making therapeutic decisions, the information about the values of the diagnostic clue is irrelevant. Such a situation occurred precisely when the usefulness of highly selective vagotomy was assessed.

## The case study of highly selective vagotomy

In a similar example to the one formulated by Worrall in his arguments against Papineau, assume that therapy *T* has been applied to people diagnosed with disease *D*. It has been ascertained that in certain patients the given therapy produced an advantageous result *R*, whereas in others the same therapy produced a disadvantageous result *¬R*. In light of these divergent results, one might assume that the final outcome of the therapy was affected by some additional factors. In such a case, the RCT research would lead only to “averaging” the divergent results. This example shows a considerable limitation of the RCT approach to research. Contrary to Worrall, however, I claim that such examples do occur in “real” medical practice. In order to support my thesis, I would like to cite the example of evaluating the effectiveness of surgical treatment of duodenal ulcer. In this case, although the RCT results seemed unequivocal, I will show how RST gives different results.

From the 1970s to the 1990s, one of the most frequently applied treatments of duodenal ulcer was highly selective vagotomy.^6^ The treatment consists in selectively severing the gastric branches of the vagal nerve, which innervates the gastrin producing area. Of all the treatments available, this one resulted in the least organ damage and produced a reasonable quality of life. Nevertheless, the surgeons did not agree in their assessments of the long-term effects of this therapy [21]. In the surgical literature, there were reports of certain cases of patients who benefited from the treatment, and of patients who did not [22–24]. The incompatible results pointed to the possible influence of some confounding factors.

The confounding factors can be postulated to be patient characteristics, some of which are closely connected with the development and degree of the disease. In this case, one could mention the following: duration of the disease, history of ulcer complications, gastric juice volume, HCl concentration, etc. These characteristics constitute a set of diagnostic indicators. Different values of these indicators could identify different states of disease *D*. Furthermore, one could also take into account characteristics such as age and sex, which are not determined by the disease, but which could be causally associated with the development and severity of the disease and, obviously, also affect treatment. All these characteristics can be labeled as attributes.

Different therapeutic results suggest that different values of these attributes affect the treatment [25, 26]. Nevertheless, the surgeons did not agree what values of these attributes affected the therapeutic results. Indications for treatment varied. Some authors considered the duration of disease to be the most important causal attribute. Others regarded the patient’s age as the basis for treatment. The opinions concerning the value of the gastric juice attributes were, however, particularly inconsistent. Various authors recognized extremely diverse values of this attribute as the justification for treatment [26, 27].

The above situation points to a very important epistemological problem: What way of obtaining empirical evidence would make it possible to provide a reliable assessment of the effectiveness of the treatment? Clinical trials could only average the results obtained without providing any directions for those asking what combination of these various attributes warranted treatment [28–30]. As Blackett and Johnston write:

when the patients with recurrence of ulcer were compared with the patients without recurrence, no preoperative factors could be identified that might be used to predict recurrence. Thus, for the two groups sex distribution, age, length of the ulcer history, previous ulcer complications, and preoperative acid outputs (basal and maximal) were very similar. Hence, contrary to some previous reports, no evidence was found that patients who are hypersecretors of acid, either basal or maximal, before operation should be treated by vagotomy.… The only factor which was found to influence the incidence of recurrent ulceration after highly selective vagotomy strongly was the surgeon who performed the operation. [25, p. 705].

When trying to establish the indications for treatment, one had to determine which of the attributes in question were causally relevant and what value of each attribute would determine the results of the therapy. Applying RST for this purpose was its first use in medicine [15].

When assessing the usefulness of highly selective vagotomy, the RST analysis initially considered 11 attributes characterizing 122 patients who had previously undergone the operation. In the assessment of the therapy’s long-term effects, this analysis employed Visick’s scale. It was demonstrated that eliminating two of the eleven attributes did not change the initial classification of the patients [15]. Thus, these two attributes were characterized as superfluous for the process of establishing the therapeutic indications.

As far as the remaining nine attributes are concerned, the RST analysis examined the extent to which the accuracy of approximation of recovery classes would decrease when the remaining attributes were eliminated. The iterative procedure began by eliminating the first attribute and determining the accuracy of approximation of the recovery classes. Subsequently, the second attribute was eliminated, and so on. Ultimately, five attributes were distinguished as the most relevant to the therapy effects. Eliminating any of these resulted in a significant decrease in the accuracy of classification of at least one of the recovery classes. Eliminating the remaining four attributes resulted in a slight decrease in the accuracy of approximation. Identifying the most important pre-operative attributes made it possible to establish the “models” of patients for each recovery class of highly selective vagotomy [15]. However, it has to be noticed that in clinical practice, one takes into account only those attributes that are of greatest causal significance. In RST analysis, remaining attributes are not incorporated into the process of a patient’s model building. The decision concerning the causal importance of attributes is completely arbitrary, which I will demonstrate using the discussed example at the end of the article.

## Epistemological assumptions of rough set theory in evaluation of clinical data

Applying RST to assessing medical data may be particularly interesting for philosophers of science concerned about the epistemology of biomedical research. It is worth investigating what RST’s major underlying assumption is.

First, we should note that many attributes characterizing the patient’s condition have values from the set of real numbers (e.g., results from the majority of biochemical tests). Nonetheless, applying the RST method requires characterizing patients in categorical sets using qualitative values. Thus, in order for the analysis to be conducted, the quantitative values need to be changed into qualitative ones which are represented by means of integers from the {0,1} set or accordingly greater. For example, for a given attribute *f*_*i*_, such that 0.3 ≤ *f*_*i*_(*x)* ≤ 7.8, *f*_*i*_ = 0, and for *f*_*i*_, such that 7.8 ≤ *f*_*i*_(*x)*, one assumes that *f*_*i*_ = 1, etc. The problem is that the transformation is arbitrary. The categorization of patients with respect to pre-therapy value attributes is not based on a difference between normal and pathological values. Furthermore, it frequently does not rely on the reference ranges that are established for diagnostic purposes. Let us consider a simple example.

Let us assume that a given attribute, *f*_*i*_, is the concentration of a certain enzyme in an organism. Assume that there is an established reference range for *f*_*i*_ from 0.3 to 0.78. In individuals diagnosed with disease *D*, the value of 0.78 has been exceeded. Attribute *f*_*i*_ can, thus, be a diagnostic clue of disease *D*.^7^ Assume also that the value of the attribute in the group in question ranges from 0.78 to 13. The example reveals the major problem with applying the RST method to assessing medical data. How can one distinguish the therapy relevant values for attribute *f*_*i*_ > 0.78? The most obvious answer would be to regard the extent of disease as the decisive factor. Consider that as the disease develops, the value of attribute *f*_*i*_ increases so that in the final stage it reaches the value of 13. In this case, however, there is no point in making use of the RST method for the purpose of establishing the rather obvious conclusion that the therapies may be less effective in later stages of the disease.

A far more interesting example could be the situation when the value of attribute *f*_*i*_ for various patients is not correlated with the disease’s stage, but rather depends on certain additional factors (unknown to the therapists). In this case, there is no clarity as to what attribute value (low, high, or very high) is beneficial for the therapy results. Obtaining such information would obviously be crucial for establishing indications and contra-indications for the therapy, but the problem is that we do not know how to distinguish the ranges of these values. Should we assume that the low range comprises the values from 0.78 to 3.4 or perhaps only 2.6? While the choice here is completely arbitrary, it nevertheless affects the results obtained in the RST analysis. This issue is considered more closely below.

Assume that a data analysis based on the RST method has shown that several patients having the same value attributes before the therapy belong to different recovery classes. If one assumes that recovery classes have been established on the basis of objective, measurable values, then there are two deterministic ways of explaining the results obtained.^8^ First, incoherent therapy results can mean that one has failed to take into consideration an unknown attribute which additionally differentiates the patients’ pre-therapeutic characteristics. The other possibility is that one has assumed erroneous value ranges for the particular attributes. The incoherence of the results obtained can thus be eliminated by modifying the value ranges of these attributes. The problem is that one does not know whether the incoherent results are due to one’s having failed to notice a causally relevant attribute or whether they are rather due to one’s erroneous differentiation of the value ranges for the attributes that have been included in one’s analysis.

Determining the influence of the particular attributes for the therapy results requires thus that two assumptions be met. Firstly, one must ascertain the value ranges for the particular attributes characterizing the patients before the therapy. Secondly, one’s analysis must take into account all attributes that are causally relevant for the therapy effects. The last of these assumptions shows that there ought to be a criterion for a preliminary selection of attributes that can be recognized as causally relevant for the therapy effects. RST alone does not provide one with such a method of attribute selection.^9^

Another problem with applying the RST method for evaluating medical data is connected with eliminating attributes that have the smallest impact on the accuracy of approximation for recovery classes. Such a procedure occurred in the case of highly selective vagotomy. Recall that when two superfluous attributes were discarded, the RST analysis identified five attributes that had the greatest impact on the therapeutic outcome. On the other hand, four other attributes were characterized as having a very small impact on the results of treatment. Thus, it was assumed that attributes having the greatest impact provided a satisfactory accuracy of approximation for recovery classes, whereas the other four could be omitted in constructing patient models [15]. Nevertheless, the term “satisfactory accuracy of approximation for recovery classes” is an epistemological term, and it, therefore, needs to be specified epistemologically. After all, eliminating attributes that are even slightly causally relevant must be appropriately justified.

It is worth noting that the problems indicated above are a consequence of defining the particular research goals that direct the performance of one’s RST analysis. The arbitrariness of establishing the value range of pre-therapy attributes, formulating explanations of contradictory therapy effects for patients displaying the same pre-therapy attribute values, as well as defining the border for satisfactory accuracy of approximation of recovery classes are not issues in the conduct of RCTs. In this type of research, however, one does not take into account the diversity of patients according to the values of pre-therapy attributes. The assumption that attributes will be equally distributed as a result of randomization renders these problems moot.

## Conclusion

In the light of the problems I have elucidated, a question arises about whether RST offers enough advantages to recommend it over classical RCT analysis. One of the indisputable advantages of RST (compared with RCTs and other statistical methods) is that it can be applied in situations in which there is information regarding a large number of attributes. Statistical methods make it possible to establish correlations exclusively for a small number of attributes. In these methods, every increase in the number of attributes considered requires increasing the number of patients examined.

Rough set theory (RST) is one of the many methods for assessing the data currently applied in medicine. At the very least, it is an interesting alternative to statistical methods for obtaining data from clinical trials, RCTs included. Both RCT studies and the RST method for assessing data make it possible to create decision algorithms. These algorithms are, however, differently justified and differ in accuracy. RCTs do not specify the rules of medical decision making for particular patients. The trial only provides information about a population, which must be subject to proper interpretation by a doctor (e.g., with the use of PICO^10^ analysis) in making decisions for particular patients. The RST method, on the other hand, allows one to determine patients’ pre-therapy characteristics, and provides clues about how to deal with particular patients who have set values of pre-therapy attributes. This is undoubtedly of great help in the process of clinical decision making.

The afore-discussed issues do not exhaust the problems connected with applying RST to medical data evaluation. They allow us, however, to identify a new area of research that ought to be examined in epistemological terms, for RST seems to be an interesting supplement to the statistical analyses that are currently applied in contemporary medicine.

## Footnotes

1 This is commonly discussed as the external validity issue; see for example [9].

2 I am using the simplest scenario of an RCT, in which the classification has a dichotomous character, and there is only one study group. Of course, it does not have to be so.

3 Of course, one can differentiate more recovery classes, e.g. improvement, no improvement, patient deterioration. For the purpose of my analyses, it is enough to differentiate two of the three mentioned.

4 For the sake of clarity, I am not discussing this issue in detail. In the specified case, one can easily calculate the accuracy of approximation for both recovery classes using the hypothetical data presented in the previous paragraph.

5 As in the previous case, I will not specify the accuracy of approximation. I will only draw attention to the fact that attribute *f*_3_ differentiated three patients: *a*_1_, *a*_5_, *a*_7_. After removing it, the doubtful region of classification increased to form set: {*a*_1_, *a*_2_, *a*_4_, *a*_5_, *a*_7_}. Undoubtedly, then, the accuracy of approximation for both recovery classes decreased.

6 Until 1990s. Later, highly selective vagotomy was replaced by pharmacological treatment.

7 I disregard the issue of determining the effectiveness of diagnostic tests for diagnosing disease D (specificity and sensitivity of diagnostic tests).

8 Yet another alternative would be that the recovery process based on the attribute is irretrievably stochastic, but I will not discuss this further.

9 That is why applying RST is in practice limited to analyzing those attributes that appear as diagnostic clues for a given disease.

10 PICO is an acronym for a technique used in EBM to frame a clinical question. Those questions should identify four issues: patient problem, intervention, comparison, and outcome.
